# Marketing and semiotic approach on communication. Consequences on knowledge of target-audiences

**Published:** 2013-03-25

**Authors:** D Borţun, VL Purcarea

**Affiliations:** * National School of Political and Administrative Studies, SNSPA, Bucharest; ** „Carol Davila" University of Medicine and Pharmacy, Bucharest

**Keywords:** marketing, semiotic, consumer, communication, cultural loading

## Abstract

Modern marketing puts the consumer and not the manufacturer in the center, the essence of the marketing approach being the conception, the projection and the making of the product, starting from the consumer towards the manufacturer; this resulting in the fact that the product’s marketing approach seems strikingly similar to the semiotic approach of the message.

In the semiotic approach, the message is a construction of signs, which, by interacting with the receiver, produces the meaning. The transmitter (the message transmitter) becomes less important. The focus is centered to the „text" and the way this is „read", the sense being born when the „reader" negotiates the „text". The negotiation takes place when the „reader" filtrates the message through the sieve of his cultural loading. A „target public" is a group which is specific to a certain Cultural Loading, a loading which deals with linguistic, logical, psychological and symbolic structures, which get out to meet the message and „negotiates" with the structures similar to it.

When we are thinking in terms of the semiotic approach, we are handling the cultural determinism of communication, using the concepts of Kuhn and Gonseth (paradigm and referential). They open a new path in the market research, in the market segmentation and knowledge of the „target audiences".

## The marketing approach

In reply to the question "What marketing is?", Kotler and Armstrong wrote: "More than any other company function, marketing deals with clients. Creating value and satisfaction for the client represents in itself the essence of modern marketing thinking and practice" [**1**]. However, this does not mean that the only purpose of marketing is client satisfaction, regardless of the manufacturer’s expenses. In the same moment, Kotler and Armstrong offer the simplest definition of marketing: "marketing is the delivery of client satisfaction at a profit." [**1**].

 Therefore, modern marketing places the consumer in the centre of attention, and not the producer, aiming at producing and selling what the consumer wants, when and where he wants it, at a price he is willing to pay. But marketing is not only about "explaining and selling"; sale and advertising is only the tip of the iceberg. Unlike sale, which only begins after the product is manufactured, "marketing begins long before the company has a product" [**2**].

In Principles of Marketing, Kotler and Armstrong define marketing as it follows: "a social and entrepreneurial process, through which individuals and groups obtain the thing they need and they desire, by creating and exchanging products and value with other groups and people" [**2**]. It is a social process – an extremely complex process, and sale is, like advertising, only one of its many functions. 

 This is the essence of the marketing approach: conceiving, designing and making the product, starting from the consumer to the manufacturer, which is the exact reverse order compared with sales, which take place from the manufacturer to the consumer. In fact, the starting point is the knowledge (as exact as possible) of needs, desires and demands existing in certain groups ("market segments"), and for building the "product’s personality" even knowledge of phantasms or false needs haunting the individuals’ conscious or subconscious is a must. In (1967), Kotler was keen on mentioning that it is not the marketers who create false needs, which "are already there before the marketers appear"; at most, they influence some desires that arise from the existing desires: "Marketers could promote the idea that a Mercedes would satisfy a person’s need for social status, but they are not the ones creating the need for social status" [**3**]. Marketing starts with the idea of identifying some needs and desires and ends up satisfying them, thus polarizing signals that the market generates, it chooses the targets to be reached, it studies the consumer’s behavior and develops strategies for: production, pricing, distribution and promotion, also using methods from psychology, sociology, anthropology etc., developing at the same time its own information system. 

In the marketing approach, a decisive importance is held by the company’s external environment, which is formed by the micro-environment and the macro-environment. The elements making up the macro-environment are the natural, the demographic, the economic and socio-cultural (religion, ethnicity, organizations, etc.) environments. When we speak about the socio-cultural environment, we firstly refer to the main cultural values of society, which are found in the people’s conceptions: image of the self (some search personal pleasures, escaping daily routine, whereas others accomplish themselves through religion, career, etc.); image of the others (the switch to an "altruistic" society, through charity, social assistance, etc); image of organizations (companies, parties, unions, state institutions, etc.); image of society (patriots defend it, reformists want to change it, the Eastern people want the living standard of the West); image of nature (some live in harmony with nature, others feel dominated and others try to master nature); image of the universe (in some areas, religious practices still exist, in others there is a permanent regression due to the search for immediate satisfactions). 

 Taking into account all the above, we may conclude that the marketing approach is strikingly similar with the semiotic approach on communication. The similarity is so striking that for the one who perceives it, it is hard not to search for its profound significance, fertile at a theoretical level.

### The semiotic approach on communication 

 In a classic paper, Introduction to Communication Sciences, John Fiske showed that in the semiotic approach the message is a construction of signs which, by interacting with the receptor, generates meaning. The focus is not so much on communication as process, but rather on communication as the generator of meaning. The sender (message transmitter) loses his importance. The focus is directed towards to the "text" and the way it is "read". "The reading" is the process of discovering the meaning that emerges when the "reader" interacts or negotiates with the "text". The negotiation takes place when the "reader" filters the message through the strainer of the cultural pattern, in terms of signs and codes that make up the message. The more we share the same codes and the same sign system, the closer the two significances attributed to the message [**4**]. 

 Therefore, the message does not occur prior to the communication process, which exists independently from the Sender-Receiver interaction, sent by the sender to the receiver, but it is an element in a structured relationship which includes, among other elements, the external reality as well as the producer/reader. Producing and reading the text are seen as parallel (if not identical); within these processes, the relationship is structured in such a way that they occupy the same place. The concomitance between communication and meaning generation, which we consider to be the essence of the semiotic approach on communication, is of paramount importance for efficient communication. Who does not think of the "Sender" and "Receiver" as co-authors of the message cannot have a professional career in Public Relations. What is the "target-audience" if not a group who has a certain cultural loading? The cultural background or the cultural loading consists of linguistic, logical, psychological and symbolic structures which greet the message and "negotiate" with its similar structures. Semiotics assess communication as generation of significance through messages – generation accomplished by either the message codifier or by the message de-codifier. Significance is not a static, absolute concept, clearly delimited in the message. It is an active process; in order to characterize this process, semioticians use verbs such as to create, to generate or to negotiate. 

 Negotiation is perhaps the most useful of them because it implies a "I-give-something – you-give-something / I-make-a-compromise – you-make-a-compromise" "meet-you-half-way" between the person and the message. Following the "negotiation" process, Meaning emerges, that is the meaning of the message – in fact, it is the message itself that emerges, as there is no message without meaning (the same way that a sign without meaning does not function as a sign). When the receiver does not have all the necessary structures for the reading, he "sees" but does not know what he sees (the so-called state of confusion/perplexity); when the receiver does not have any of these structures, the message directed at him "is lost in the cosmic obscurity". That is why, in the Public Relations profession, the engineering of effective and efficient communication is mandatory in the research phase, in order to get to know the cultural background of the target-audience.

 This is how we can graphically represent the generation of significance (of the birth of meaning) after the negotiation process between the message structure and internal structures of the subject: 

**Fig. 1:**
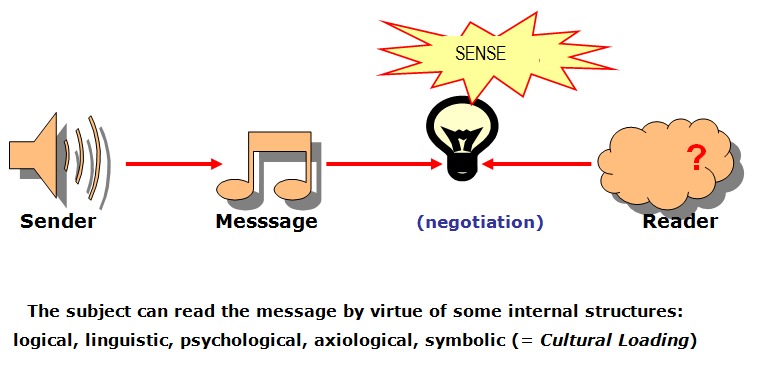
The birth of meaning in the semiotic approach of communication

### Consequences of target-audiences on knowledge

 When we think in the terms of semiotic approach, which highlights constant, biunique interactions between the message "producer" and the reference system, between him and the "reader", we deal with the cultural determinism of communication, using the concepts of Kuhn and Gonseth (paradigm and referential respectively).

 The connection between inter-individual and cross-cultural communication becomes obvious the moment we understand culture in the paradigm of cultural anthropology – for example, as E.B. Tylor, T. Parsons or Chombart de Lauwe define it. In the introductory study of the volume Images de la culture, called "Systemes de valeurs et aspirations culturelles", Paul-Henry Chombart de Lauwe classified the approaches on culture as it follows: 1) culture as development of the individual within society, 2) cultures belonging to societies or to private social environments and 3) the issue of developing an universal culture. It is obvious that out of the three approaches, the only one which does not involve a prior assessment and which does not necessarily lead to a hierarchy of cultures (societies, groups or individuals) is the second one. It will also be the privileged referential of this paper, as it best suits its objectives. 

 Stressing the role of infrastructure generating aspirations and value systems, Chombart de Lauwe considers that "a culture is marked by a series of patterns, guiding images, representations to which all members of a society relate to in their behaviors, work, role and social relationships". He draws attention to the equal importance that techniques, space organization, production and work or consumption have. 

### The cultural paradigm

 The concept of "cultural paradigm" has been used increasingly more over the last four decades, both in social philosophy, and in anthropology, psychology and sociology [**5**]. It entered these fields in the form of "concept translation", borrowed from the philosophy of science, where it was imposed by the American philosopher Thomas S. Kuhn. He was the one to realize that theories on the nature of science and the purpose of research in natural sciences do not concord with the scientific practice, as it ensues from the history of science. In practice, he says, the behavior of scientists deviates from the canons which define scientificity and even rationality (canon which we encounter both in science philosophy and in current mentality). Positivists, and even K. Popper (adversary to logic empirics), considered that science differs from speculation by testing – either as a confirmation of the theory (Carnap), or as an invalidation ("falsification") of testing (Popper). For them, the central concept in characterizing the nature and the dynamics of science is that of "scientific theory" and the differentiation criterion between science/ non-science is testability. For Kuhn, the core concept is the one of paradigm and the criterion is problem solving. 

 Paradigms are patterns of scientific practice that can be encountered in classical scientific works and especially in handbooks and treaties; they are at the bottom of a disciplinary group education (physicians, chemists etc.). Based on these, the one who educates himself learns to formulate and to solve new problems. Paradigms are therefore "exemplary scientific accomplishments which, for a period of time, offer model problems and solutions to a community of practicians" [**6**]. 

 Unlike the knowledge contained in the abstract assertions of theory and in the general methodological rules, knowledge in paradigms is a tacit knowledge. Paradigms guide the members of the scientific group towards solving new problems, without them being aware of the paradigm every step of the way. They apply it – sometimes even creatively – but without being able to speak about it in general statements. 

 Their collective character results from the quasi-conscious character of paradigms. Although the birth of a paradigm is usually linked to the name of a great thinker (Ptolemy, Newton, Franklin or Einstein), it is never the making of a single man. 

 The fact that the members of the disciplinary group share a paradigm explain the fact that they communicate almost perfectly and without major difficulties; moreover, it explains the unanimity of professional judgments. This does not happen however with scientists who share different paradigms, as paradigms are incommensurable (cannot be compared, because there is no common "unit of measure").

 The incommensurability of paradigms originates from the following: i) they imply incompatible presuppositions regarding the basic entities of the studied domain and regarding their behavior; ii) they imply different criteria of delimiting "real" problems and "legitimate" solutions; iii) the observations which scientists make on the same reality are also incommensurable. 

**Fig. 2:**
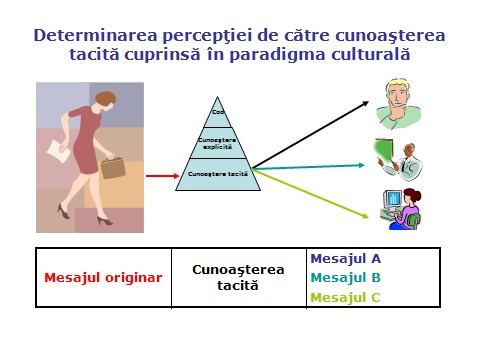
Filtering the message of tacit knowledge in the paradigm

 How can the incommensurability of observations be explained? Although they look "in the same direction and from the same standing point" (Kuhn), although the constitution of the sensory apparatus is the same, researchers will perceive different things. This happens because of the tacit knowledge in paradigms; it interposes on the stimulus-perception circuit (**Fig. 2**). 

 Thus, a "communication fracture" (Kuhn) appears; the adepts of a paradigm cannot convince the adepts of a competitive paradigm of the superiority of their viewpoint and they will not be able to understand and accept the other’s point of view. The arguments of the two parties will be circular (they can be understood and accepted only by the researches who are already working in the same paradigm). 

 It suffices to replace Kuhn’s concept with the one of "cultural paradigm" in order to realize that the limits of communication between scientists are valid for the communication between any human groups – since any group can be considered a cultural or sub-cultural community (ethnic communities, social classes, professional guilds, political parties, etc.) [**7**]. It suffices for two rival (in other words, competing for the same realm of reality) paradigms to exist in order for obstacles to appear in communication. 

 We shall define the cultural paradigm as a constellation of values, beliefs and methods (including "techniques" of problem formulation) shared at a certain moment by the members of a community. 

 One moment after, we shall realize that all of Kuhn’s observations regarding "disciplinary groups" remain valid: 1) the partisans of rival paradigms speak of different things, even when they look "from the same standpoint" and "in the same direction"; 2) competition between rival paradigms is not solved with arguments or by resorting to "facts"; 3) the adepts of rival paradigms disagree with respect to "the really important problems"; 4) communication between them is always partial; v) the adepts of rival paradigms are in different worlds (they see different things, in different correlations); 5) absolute communication is possible only inside the same paradigm; 6) the switch from one paradigm to another can occur for various reasons, which are not related to logical demonstration or empiric "proofs". 

 The idea of "paradigm" was developed, in culture and civilization studies, in consonance with the great trends of science and philosophy, by the French thinker Edgard Morin, in The lost paradigm: human nature [8,9]. Maruyana defines four epistemological typologies which correspond to different ways of perception, causality and logic: a) the homogenizing/ classifying/ hierarchical type; b) the atomist type; c) the homeostatic type; d) the morphogenetic type. 

 Each of the types above engenders a mindscape which colours any creation in the sphere of knowledge, esthetics, ethics and religion. 

 Through its radicality and universality, Maruyama’s conception resembles that of Michel Foucault, who speaks of epistemes as of likelihood conditions of the cognitive field accesible to a culture: "the array of relations hat unite, at a certain moment, those discourse practices that generate epistemological figures, sciences and virtually formulated systems [of knowledge]" [**10**]. Foucault postulates the uniqueness of the episteme within a culture. But in "open societies", a culture presents itself as a "game of paradigms", as a network of paradigms, sub-paradigms and meta-paradigms. There can be no question of a "unifying paradigm", but the existance of some dominant paradigms and some dominated paradigms is obvious. 

 If we find in Morin the same radicalims as in Foucault, the former will not however postulate the uniqueness of a certain paradigm within a culture (in an era or in a community). Morin talks of "large" and "small" paradigms, of "adversary", "intolerant" paradigms etc. In Edgar Morin, a "large paradigm" controls both the theories and the reasoning, as well as the cognitive (intectual and cultural) field where theories and reasoning form. It controls even the epistemology which controls the theory, even the practice to which theory relates to. The individuals of a community know, think and act according to the paradigm that their culture has written in them. We apparently stand no chance of changing their thinking and acting strategies. 

### Tacit communication 

 The only realistic solution is to use tacit communication, which could reach for the "self-image" of groups and individuals from various social groups, in order to trigger the change of some of the current cultural paradigm’s presuppositions, in particular of those which generate perceptions, representations and value-attitude couples, which, in their turn, generate contra-productive behaviors (which oppose the purposes of modernization). In essence, it is the art of talking about something while leaving the impression that you talk about something else. 

 To those who will cry resentfully "This is manipulation!" we reply: 1) manipulation "abhors vacuum", because if we do not manipulate, others will; 2) assuming nevertheless that no one is manipulating them, people would manipulate themselves- which they do, "day after day, hour after hour, and in mass", by virtue of desiderative thinking, of inauthentic thinking (Erich Fromm) and of the "voluptuousness of self-deception" (Jean-Francois Revel); 3) manipulation is not an evil in itself; it can be good or bad, depending on the purpose; 4) nothing great can be accomplished without manipulation – from bringing up a child to educating a people -, and we do not speak of the modernization of the Romanians, a much more complex and difficult task then education. Conclusion: he who fends himself from manipulation is manipulating himself, but against him; he is remain a «part of the problem and will never be "part of the solution". 

 A pre-condition for success is the positive knowledge of value-attitude couples which are at work in today’s Romanian society, knowledge that will be ensured by mixing theoretical approaches with national-scale sociologic research, carried out by a specialized institute. 
